# Efficacy of Autogenous Dentin Biomaterial on Alveolar Ridge Preservation: A Randomized Controlled Clinical Trial

**DOI:** 10.1155/2023/7932432

**Published:** 2023-12-27

**Authors:** Afnan Abdulkareem Hussain, Ahmed Fadhel Al-Quisi, Ali A. Abdulkareem

**Affiliations:** ^1^Department of Periodontics, College of Dentistry, University of Baghdad, Baghdad, Iraq; ^2^Oral and Maxillofacial Surgery Department, College of Dentistry, University of Baghdad, Baghdad, Iraq

## Abstract

**Background:**

After tooth extraction, alveolar bone resorption is inevitable. This clinical phenomenon challenges dental surgeons aiming to restore esthetic and function. Alveolar ridge preservation can be applied to minimize dimensional changes with a new socket grafting material, an autogenous dentin graft, produced by mechanically and chemically processing natural teeth. This study assessed the safety and efficacy of using autogenous dentin biomaterial in alveolar ridge preservation.

**Materials and Methods:**

Patients with nonrestorable maxillary anterior teeth bounded by natural sound teeth were included in this study. After a detailed clinical and tomographic examination, eligible participants were randomly allocated into two groups. The control group had spontaneous healing of extraction sockets. The study group had their extraction sockets filled with autogenous dentin biomaterial after processing their extracted retained roots with the KometaBio device. Standardized cone beam computed tomography (CBCT) scans were repeated four months later. A full-thickness mucoperiosteal flap reflection was achieved under local anesthesia to get core biopsies for histomorphometric analysis, and dental implants were placed at the same session.

**Results:**

A total of 32 eligible patients were included in this study (*n* = 16 in each group). Both groups had significantly higher facial soft tissue thickness after four months than baseline (*p* < 0.05). However, the study group showed statistically significant lesser dimensional changes than the control group according to the standardized CBCT scans. Furthermore, core biopsies confirmed an excellent remodeling of the autogenous dentin biomaterial in the study group. In comparison, only new thin bone trabeculae-filled sockets were in the control group.

**Conclusion:**

Autogenous dentin graft can be safely and successfully used for alveolar ridge preservation. Optimal graft remodeling histologically, better ridge dimensional stability, and uneventful wound healing support its clinical application. This trial is registered with TCTR20220615002.

## 1. Introduction

Dimensional changes in the alveolar bone after tooth extraction are well-known [1, 2]. These dimensional alterations happen within the first eight weeks after dental extraction, with the buccal portion of the socket being the most affected part [2, 3]. Various studies revealed that alveolar bone loss was 11% to 22% of alveolar bone height and 29% to 63% of alveolar bone width over the first 12 months following tooth extraction, whereas two-thirds of the ridge was lost during the first three months [4, 5] in the esthetic anterior maxillary region, where a thin buccal bone plate significantly affects the dimensional stability of the newly extracted socket [2, 6].

A thin, atrophied alveolar ridge may pose challenges in achieving favorable esthetics and long-term success with dental implant therapy in this critical zone. When immediate implant placement is not recommended, alveolar ridge preservation (ARP) techniques are used to minimize the reduction of the alveolus dimensions after tooth extraction. Most of these techniques include applying space-preserving biomaterials [7, 8], such as xenogeneic, allogeneic, or synthetic bone substitutes, depending on their osteoconductive abilities, mainly with or without using barrier membranes [7]. An optimal bone graft should possess osteoconduction, osteoinduction, and osteoproliferation characteristics for the best outcomes [9]. Since 2008, Kim et al. developed and used an autogenous dentin graft in clinical applications [10–12].

Histologically, human dentin and enamel comprise 45% organic material and 55%inorganic structure, primarily hydroxyapatite [13]. The organic component mainly includes type I collagen and bone morphogenic proteins (BMP). The dentin extracellular matrix comprises approximately 90% type I collagen [14]. The organic and inorganic components of the human teeth and bones are highly similar.

Autogenous dentin biomaterials (ADB) have osteoinductive and osteoconductive properties with organic and inorganic ingredients, making them suitable as a grafting substitute for alveolar ridge preservation ARP [12, 15]. Despite much research in recent years evaluating the effectiveness of the ADB in ARP by using linear measurements in CBCT's sagittal sections at two-time points [16–20], no one of these previous studies limiting the inclusion criteria into single nonrestorable retained root with intact bony walls in the maxillary nonmolar sites bounded by natural sound teeth to eliminate the possible confounding factors that may influence the results. Furthermore, establishing a standardized point for the CBCT linear measurements is mandatory for accurate assessments. This study is aimed at evaluating the role of autogenous dentine biomaterials in preserving the alveolar ridge after extraction of a single retained root in the maxillary nonmolar sites by clinical, tomographical, and histological examinations with a surgical stent as a reference point [21]. We hypothesized a difference between spontaneous healing and preserved extraction sockets, while the null hypothesis is that there is no significant difference between spontaneous healing and preserved extraction sockets with ADB in terms of dimensional changes and healing characteristics.

## 2. Methodology

### 2.1. Study Design and Center

The study was conducted at the University of Baghdad's College of Dentistry's Department of Periodontics from June 2022 to April 2023. It was designed as a double-blind, randomized, parallel arm-controlled clinical trial and followed the Consolidated Standards of Reporting Trials (CONSORT) guidelines [22]. The research protocol was registered on https://www.thaiclinicaltrials.org/show/TCTR20220615002 on 15 June 2022 with the identification number (TCTR20220615002), and research protocol and informed consent template requests were approved by the Research Ethics Committee, College of Dentistry University of Baghdad Institutional Review Board on 17 June 2022 (Ref. number 525).

### 2.2. Eligibility Criteria and Recruitment

Adult patients who needed extraction for maxillary nonmolar teeth, bounded by sound teeth, and were interested in participating in the study were screened for eligibility. The criteria for exclusion were as follows: (1) Patient diagnosed with periodontitis based on clinical and radiographic examination—clinical attachment loss was detected interdentally at two or more nonadjacent teeth, or the presence of clinical attachment loss (CAL) was ≥3 mm, associated with probing pocket depth (PPD) >3 mm buccally or orally, detectable at ≥2 teeth [23]; (2) acute infection at the site of interest; (3) general medical condition that may interfere with dental surgery and dental implant placement, for instance, noncontrol diabetes, heart disease, and osteoporosis; (4) pregnant or lactating women; (5) history of any surgical treatment for the accused tooth; (6) subjects with habits such as smoking, tobacco chewing, or alcohol; (7) patients with a history of radiotherapy or metabolic diseases, immunosuppressive agents, or use of systemic corticosteroids or intramuscular/intravenous/bisphosphonates; (8) absence of the buccal or palatal bone plate as checked in the cone beam computed tomography

During the screening visit, all patients signed informed consent before the clinical and tomographic examination, after informing them about the study's purpose, design, and timeline, with the foreseeable advantages and possible hazards associated with their participation.

### 2.3. Primary and Secondary Outcomes

The study assesses the clinical and tomographic results of alveolar ridge preservation after four months of surgery. The primary outcomes are linear phenotypic dimensional changes, which were assessed by measuring the midfacial and midpalatal bone and soft tissue thickness at baselines and after four months of healing at two points in millimeters (mm). The secondary outcomes include changes in the vertical alveolar bone of the socket buccal wall in (mm). Furthermore, histomorphometry will be conducted to evaluate new bone formation in both groups four months after the initial surgery.

### 2.4. Sample Size Calculation

G-power software (V.3.1.9.7, Kiel, Germany) was applied to calculate the sample size depending on the summary statistics of the mean changes of the horizontal alveolar bone width (1.30 ± 0.63 and 2.25 ± 1.71) for study and control groups, respectively, obtained from the pilot study and used to calculate the effect size, which was equal to 0.7038. This value was used based on two dependent means (two-tailed) with a significance level alpha set at 5% and power at 95%. This resulted in 16 subjects for each group with an added 10% dropout rate.

### 2.5. Randomization and Masking

Eligible patients were randomly assigned to alveolar ridge preservation with ADB (study group) or unassisted healing (control group) using permuted block randomization with Microsoft Excel 2016 to achieve a 1 : 1 allocation ratio for the groups. All patients were equally prepared and operated on by the first investigator. At the same time, the second researcher was responsible for allocating participants into groups. After completing the dental extraction and processing of the extracted teeth, the second researcher revealed the intervention allocation to the investigator according to the generated sequence. All outcome assessors and biostatisticians were masked to treatment type. However, blinding of the investigator was not possible.

### 2.6. Clinical Procedure

All the eligible patients received a professional oral hygiene session two weeks before the initial surgical intervention. Chlorhexidine, 0.2% mouth rinses (Periokin, Spain), was prescribed twice daily for two weeks. To assess their periodontal status, plaque index, probing depths, gingival recession, and bleeding on probing (BOP) were evaluated at six sites (midfacial, mesiofacial, distofacial, midpalatal, mesiopalatal, and distopalatal) around the tooth to be extracted as well as on the adjacent teeth.

**Figure 1:**
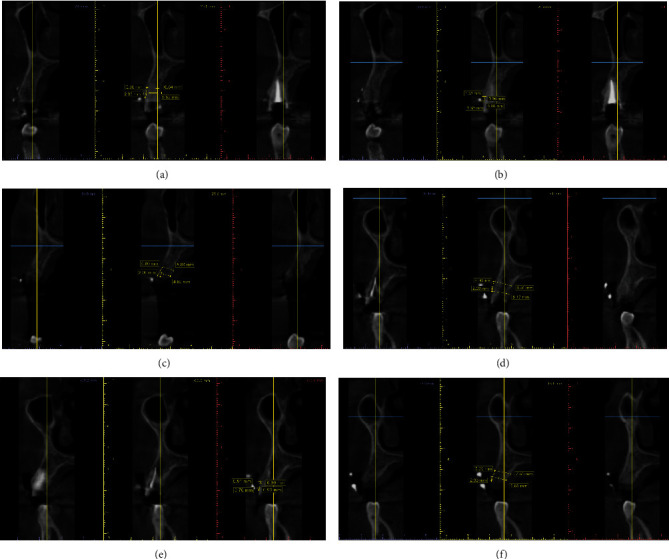
Tomographic measurements of the alveolar ridge width, gingival biotype, and buccal bone plate thickness. (a) Nonrestorable upper right first premolar. (b) Preoperative assessments of the gingival biotype and buccal bone plate thickness. (c) 4 months after socket healing (control group). (d) Nonrestorable upper right first premolar. (e) Preoperative assessments of the gingival biotype and buccal bone plate thickness. (f) 4 months after socket healing (study group).

The diagnostic stone cast was made after an intraoral impression with a silicone impression material (Zhermack Zeta Plus, Italy). The surgical stent was fabricated using a vacuum former and Biostar acrylic sheet 1.0 mm thick (JINTAL, China). Two points (2 and 5 mm from the gingival margin) were made at the implant placement site and marked on the stent by drilling holes with a diameter of 1.0 mm. for buccal/lingual alveolar ridge measurement. Furthermore, these points were filled with gutta-percha for use during the preoperative tomography to provide radiopaque landmarks indicating the locations for comparative tomographic ridge width measurement. All scans were achieved by Kavo OP 3D PRO (Biberach, Germany) using fixed parameter voxel size, 66 kV and 9.9 mA. Scans were exported in DICOM format to 3D viewer software (Blue Sky Plan 4.7.2, Blue Sky Bio, USA), as shown in Figure 1.

The facial gingival thickness was assessed using an endodontic reamer (no. 40) fitted with a rubber stopper and inserted perpendicularly at 2 and 5 mm through the guidance holes in the stent under the influence of the topical anesthetic gel. When resistance felt, the rubber stopper was repositioned in contact with the gingiva and fixated in place with a flowable composite. The resultant distance was measured with a digital caliper with 0.01 mm sensitivity.

All dental surgeries were carried out under the administration of local anesthesia. The technique of flapless tooth extraction was performed using periotomes and forceps to minimize surgical trauma. All alveolar sockets were gently curetted, irrigated with normal saline, and inspected for bony wall defects. According to the developers' instructions, the extracted retained roots were prepared immediately after extraction with the KometaBio device (KometaBio, Smart Dentin Grinder, USA). First, a high-speed handpiece removes all decay, artificial material, gutta-percha, and debris from the patient's extracted roots so that only the clean tooth root remains. The KometaBio tooth grinder was used to crush the roots containing dentin.

**Figure 2:**
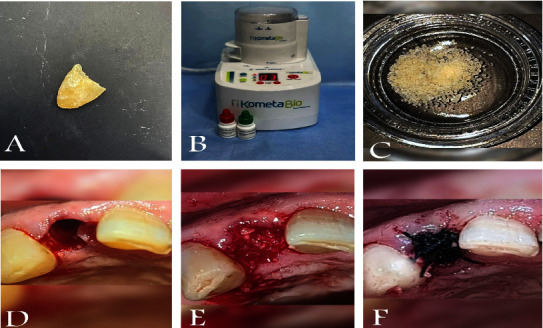
Clinical steps of graft harvesting, preparation, and insertion. (A) Root of an extracted tooth. (B) KometaBio grinding device. (C) Graft preparation. (D) Extraction site. (E) Grafting. (F) Suturing.

The resulting root dentin particulate material underwent a 3-step first: dentin cleanser solution was poured into the dish with the particulate for 5 minutes at room temperature. After that, a sterile gauze was used to dehydrate the solution. Next, the phosphate-buffered saline (PBS) was poured into the dish, covering the particulate completely. A sterile instrument was used to mix the particulate and dehydrate it using a new sterile gauze. Repeat this step with the PBS solution again. This step is essential to neutralize the pH levels. Finally, the ADB with particle size 300-1200 microns is ready for immediate grafting. The extraction sockets in the study group were filled with ADB powder and then covered with gel foam (Roeko Gelatamp, Germany). Finally, figure eight suture was used to stabilize the extraction sites in both groups (Figure 2).

Four months after extraction, standardized CBCT scans (with the same old stent inside the patient's mouth) were taken to evaluate alveolar bone changes at the sites of interest. The second surgical intervention was performed under local anesthesia with an incision and reflection of the full-thickness mucoperiosteal flap. Core biopsies were harvested for histological analysis at implant osteotomy sites using 2.6 mm inner/3.6 mm outer diameter trephine burs in 10 mm depth in the planned implant axis under copious irrigation of normal saline (Komet Dental, Lemgo, Germany) followed by implant placement from Bionnovation Implants Biomaterial (Brazil) and suturing.

**Figure 3:**
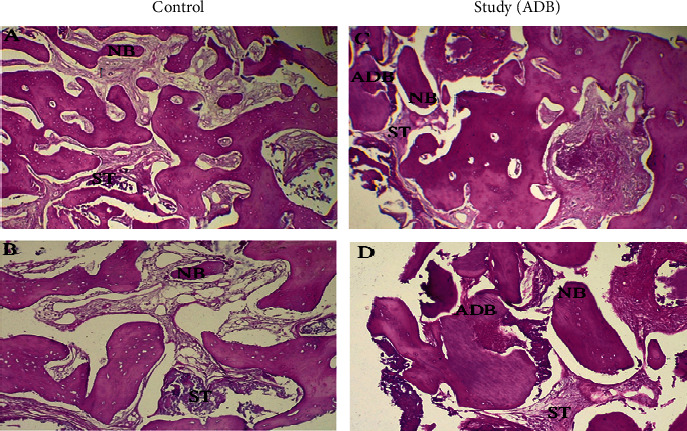
Qualitative histological analysis. Representative microscopic images ((A, C) 40x and (B, D) 100x magnifications) with H&E staining of histological sections. Abbreviations: NB: new bone; ADB: autogenous dentin biomaterial; ST: soft tissue.

Core biopsy samples were fixed in formalin, embedded in paraffin wax, and sectioned for histological examination. Afterward, hematoxylin and eosin (H&E) staining was carried out, and blind quantitative histological analysis was performed by a trained specialist using the ImageJ® v1.52a software (U.S. National Institutes of Health, Bethesda, Maryland, USA). ImageJ® scan downloaded from http://imagej.nih.gov/ij/ software individually adapted for histomorphometry (Figure 3).

### 2.7. Follow-Up and Postsurgical Care

Participants were given specific instructions to avoid eating on the surgical site and to avoid vigorous mouth rinsing or drinking hot drinks. Participants were encouraged to brush gently around the surgical site two weeks after surgery, while ordinary tooth brushing for other parts of the dentition should be maintained from the first day after surgery. Amoxicillin (500 mg TDS) or azithromycin tablet (500 mg O.D.) in case of penicillin allergic with Ponstan forte tablets 500 mg as required were prescribed for five days for both groups. Additionally, a mouthwash of 0.2% chlorhexidine has been prescribed for rinsing twice daily for 2 weeks. Sutures were removed 7 days after the surgery.

### 2.8. Statistical Analysis

Data were analyzed using the SPSS statistical tool (SPSS®. 26.0; SPSS, Chicago, IL, USA). The normality of data distribution was assessed using the Shapiro-Wilks test. Paired sample *t*-test and Related-Samples Wilcoxon Signed Rank Test were used to compare baseline and 4-month measurements. Independent sample *t*-tests and Mann–Whitney *U* tests were used to compare measurements between the control and the study groups. The significance level was set at *p* value <0.05.

## 3. Results

### 3.1. Group Characteristics

**Figure 4:**
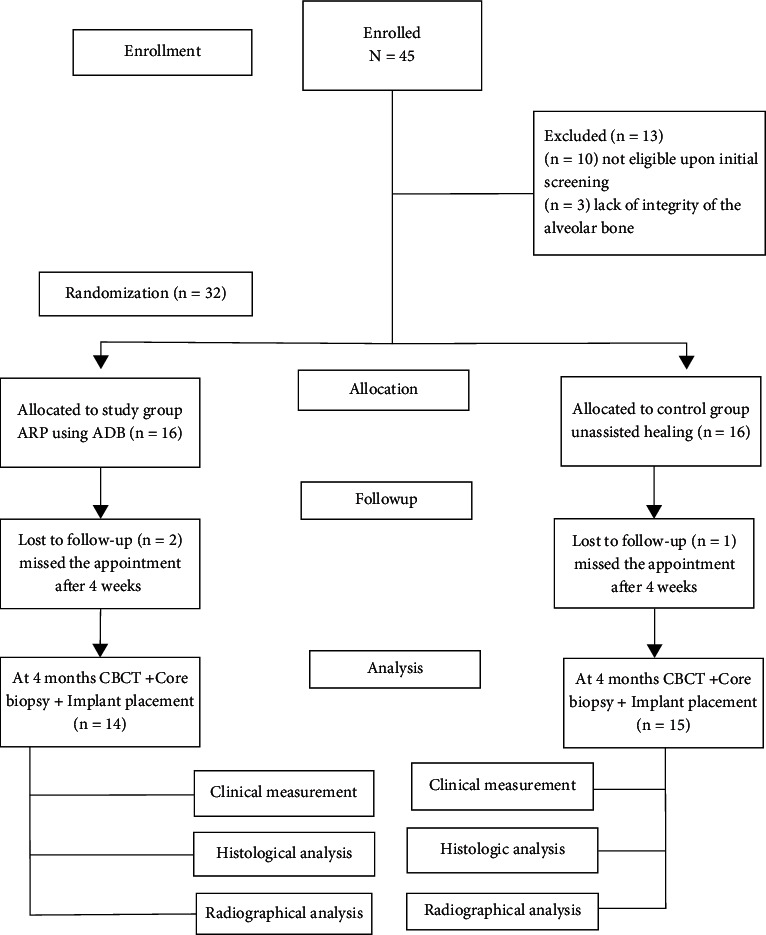
Flow chart of the study.

Forty-five patients were initially screened. Ten were not eligible, as three had a bony defect in the alveolar buccal wall revealed after dental extraction; three participants were lost to follow-up after the initial surgery, resulting in a final study sample of 29 participants who completed the study (11 men and 18 women). It was randomly distributed into two groups, 15 in the control group and 14 in the study group, as detailed in Figure 4.

Before dental extraction, it was confirmed that the individual had proper oral hygiene by assessing entire mouth plaque and bleeding on probing scores that were less than 10% of the sites. The demographic distribution of the data between the groups showed statistically insignificant differences, as shown in Table 1.

### 3.2. Clinical and Tomographical Outcomes

In both control and study groups, facial soft tissue thickness was significantly increased at 2 mm and 5 mm after 4 months from the initial surgery compared to baseline records inside each group. Furthermore, there was a significantly lower ridge width at 5 mm and 10 mm after 4 months compared to baseline records, as shown in Tables 2 and 3.

When comparing the net change of the clinical variables between the groups, it did not reach the statistical significance level despite the study group achieving fewer changes in the facial soft tissue thickness. However, tomographical results showed that the study group achieved significantly fewer alveolar bone dimensional changes in the buccolingual direction at 2 mm and 5 mm reference points than the control group, as shown in Table 4.

### 3.3. Histological Outcomes

Histological examination of the study group revealed some remnants of dentin materials surrounded by new mature bone. In contrast, the control group had only a few new thin bone trabeculae-filled sockets containing large osteocytes lined by osteoblasts. Osteoclast is also present in some areas, which means continuous bone remodeling (Figure 2).

Based on the histomorphometric overview, the study group demonstrated a significantly higher surface area of trabeculae bone than the control group (*p* < 0.01). Meanwhile, the study group had a significantly lower surface area of bone marrow than the control group (*p* < 0.01). However, the two groups had no significant difference in total bone area (*p* > 0.05), as shown in Table 5.

## 4. Discussion

Tooth extraction triggers a series of biological reactions influenced by both the local inflammatory response that occurs after the surgery and the lack of masticatory stimulation on the periodontium. This leads to a disruption of the homeostasis and structure of the periodontal tissues. The extraction socket healing process begins with oral tissues' reorganization, followed by the proliferation and maturation of these tissues; this healing process ends with dimensional changes to the alveolar bone and gingival tissues [24, 25].

Following tooth extraction, the hard and soft tissues under dimensional change can significantly influence the esthetic and functional outcomes of the implant-supported prostheses, especially in the anterior maxillary area [26, 27].

Based on the systematic review findings, the ADB can be an alternative, cost-effective, and sustainable biomaterial for alveolar ridge preservation [21].

Different graft materials are currently available for alveolar ridge preservation. However, ADB can eliminate the inherent risks associated with these materials, like heterogeneity, immunogenicity, and cross-infection, that may be associated with allografts and xenografts [12]. Furthermore, ADB surpasses autogenous bone grafts as they do not require a second surgery, resulting in less morbidity and high predictability [4].

This study proved the effectiveness of using ADB powder during ARP procedures. Clinical, tomographical, and histological data demonstrate favorable postextraction socket preservation utilizing an autogenous dentin grafting material.

This study indicated that there was a significant increase in facial soft tissue thickness at 2 mm and 5 mm after 4 months compared to baseline (*p* < 0.01) in both the control and study groups and no significant difference in thickness between the two groups (*p* > 0.05). This finding is consistent with the Chappuis et al. study, which stated that about 50% of soft tissue dimensional alteration occur within the first two weeks of healing. These changes are directly related to the underlying buccal bone plate thickness. In a thick periodontal phenotype, the thick bony wall provides a suitable environment, favoring the ingrowth of bony tissues from the surrounding bony walls [28]. This contrasts with thin periodontal phenotypes; rapid resorption of the thin buccal bone plate favors soft tissue ingrowth into the socket seven times more than bony healing and leads to spontaneous soft tissue thickening [29]. Some fibroblasts differentiate into myofibroblasts, which stabilize wound margins and may be involved in the thickening phenomenon. Other studies have shown a trend toward soft tissue thickening following tooth extraction [30–33].

On a molecular level, eight weeks after tooth extraction, the soft tissue thickening is associated with increased endothelial cell density, bone morphogenetic protein-7, and osteocalcin expression [34]. Therefore, the molecular and cellular mechanisms that control new bone formation may also influence soft tissue thickening [34–36].

Despite multiple methods for measuring the alveolar ridge dimensions [37], CBCT remains the gold standard for measuring residual alveolar ridge width when planning dental implant treatment due to its high sensitivity and specificity [38].

The KometaBio device grinds, disinfects, and prepares extracted teeth to obtain ADB ready-to-use. ADB acts as a resorbable scaffold and space-maintaining device to facilitate healing in ARP procedures. This study showed that after 4 months of using ADB for ARP, ridge width was significantly decreased at 2 and 5 mm compared to baseline records, with a nonsignificant reduction in the buccal and palatal heights compared to the baseline records.

These unavoidable changes in the alveolar bone dimensions occur primarily within the first two weeks of the healing process. Despite efforts, postextraction ridge resorption can only partially be prevented, as the literature states [39]. Bone modeling in single extraction sites mainly occurs in the central aspect of the buccal bone wall; at the same time, the periodontal ligaments of adjacent teeth maintain mesial and distal bony wall thicknesses.

In a meta-analysis assessing dimensional changes of alveolar sockets after tooth extraction without preservation, mean horizontal bone resorption of 3.87 mm was reported, along with mean buccal and lingual losses of 1.67 mm and 2.03 mm, respectively [40].

However, the current study showed that ADB achieved statistically significant, much fewer dimensional changes in the alveolar bone dimensions (horizontal and vertical) than the control group. These results aligned with a meta-analysis that stated an alveolar ridge dimension decrease of 3.79 ± 0.23 mm horizontally and a reduction of 1.24 ± 0.11 mm on the buccal side six months after extraction [24]. Elfana et al. showed that ARP did not prevent dimensional changes in the extraction socket despite using two types of AWTG or ADDG in their study. However, the dimensional changes in the preservation group were much lower compared to the values previously reported for extraction without alveolar ridge preservation [17].

Bone histomorphometry remains the only method to analyze bone at the tissue and cell levels despite the development of noninvasive approaches [41, 42]. Under histologic observations, it has been confirmed that ADB demonstrates biological compatibility and does not cause any significant inflammatory reaction. This is evidenced by the formation of vascularized mature bone trabeculae and soft tissue matrix, which proves their osteoconductive function in bone formation. These findings have been observed in various clinical scenarios where ADB was utilized [43, 44].

The histomorphometric analysis further revealed higher quantities of newly formed bone trabeculae and fewer graft remnants of ADB. The histomorphometric analysis reveals the following findings in core biopsy; the average amount of newly formed bone was significantly higher. Surface area of trabeculae bone in the study is compared to the control group, and there was a significantly lower surface area of bone marrow in the study compared to the control group. These results of the histological analysis were superior when compared with literature using different preservation procedures, with only restricted graft remodeling in newly formed hard tissues several months after ARP [7].

Barone et al. utilized a combination corticocancellous porcine bone and collagen membrane for alveolar ridge preservation. After seven months, core biopsies showed that the connective tissue comprised about 36.6% of the specimen, with only 35,5% of the newly formed bone and 29.2% of the remaining graft materials [45]. Meanwhile, Artzi et al. utilized porcine-based grafting material for ARP; core biopsies following nine months showed that the newly formed bone constituted about 46.3% of the specimen with 30.8% of the remaining grafting material [46].

These findings suggest a faster and more efficient turnover of ADB graft particles in this study compared to literature reports on xenogeneic materials utilized in ARP.

Histomorphometric analysis of this study proved that ADB has the ability to be osteoinductive and osteoconductive, with graft particles rapidly turning. Compared with other biomaterials, this novel graft material ADB is less costly [47], well-accepted by patients, and produces fewer adverse reactions than some xenografts [48]. Moreover, a recent systematic review implies that the prepared chairside autogenous dentin blocks could be a viable alternative to other established bone augmentation techniques for staged ridge augmentation [49].

### 4.1. Study Limitations

One restriction of this study is that only one type of graft was utilized, and there was no comparison with other types of bone grafts. Only nonmolar teeth sites of the upper jaw were included in this study. Additionally, patient-reported outcomes, such as pain scores, were not evaluated.

## 5. Conclusions

Autogenous dentin graft can be safely and successfully used for alveolar ridge preservation. Optimal graft remodeling histologically, better ridge dimensional stability, and uneventful wound healing support its clinical application.

## Figures and Tables

**Table 1:** Distribution of the study variables between the groups.

Factor	Group	*N*	Mean ± SD	*p*-value^∗^
Age	Control	15	35.00 ± 11.31	0.766
	study	14	36.29 ± 11.72	

Plaque index	Control	15	6.98 ± 1.96	0.264^§^
	study	14	7.76 ± 1.89	

Bleeding on probing	Control	15	6.41 ± 1.87	0.552
	study	14	6.82 ± 1.77	

Baseline ridge width at 2 mm in CBCT	Control	15	7.94 ± 1.17	0.988
	study	14	7.93 ± 0.99	

Baseline ridge width at 5 mm in CBCT	Control	15	8.68 ± 1.10	0 .334
	study	14	9.06 ± 0.97	

Baseline buccal bone plate thickness in CBCT	Control	15	0.72 ± 0.15	0.428
	study	14	0.78 ± 0.24	

Baseline facial gingival thickness CBCT	Control	15	0.85 ± 0.23	0.476
	study	14	0.92 ± 0.30	

Baseline keratinized tissue width	Control	15	4.85 ± 1.12	0.255
	study	14	4.35 ± 1.17	

^∗^Independent sample *t*-test. ^§^Mann–Whitney *U* Test.

**Table 2:** Comparing the changes of the soft tissue thickness at the facial and palatal site and ridge width at baseline and after 4 months of follow-up in the control group.

Indicator	Comparison	Mean (mm) ± SD	*p* value^∗^
Facial soft tissue 2 mm clinical	Baseline	1.40 ± 0.66	0.0001
	4 months	3.13 ± 0.73	

Facial soft tissue 5 mm clinical	Baseline	2.17 ± 1.02	0.002
	4 months	3.40 ± 0.92	

Ridge width 2 mm with CBCT	Baseline	7.94 ± 1.17	0.0001
	4 months	4.39 ± 0.70	

Ridge width 5 mm with CBCT	Baseline	8.68 ± 1.10	0.0001
	4 months	6.48 ± 0.91	

Vertical bone height changes in the buccal bone plate by CBCT	Before dental extraction	11.32 ± 2.83	0.20
	Four months after dental extraction	10.48 ± 2.50	

Vertical bone height changes in the palatal bone plate by CBCT	Before dental extraction	11.53 ± 2.85	0.25
	Four months after dental extraction	10.82 ± 3.04	

^∗^Paired sample *t*-test.

**Table 3:** Comparing the changes of the soft tissue thickness at the facial and palatal site and ridge width at baseline and after 4 months of follow-up in the study group.

Indicator	Comparison	Mean (mm) ± SD	*p* value^∗^
Facial soft tissue 2 mm clinical	Baseline	1.82 ± 0.48	0.002
	4 months facial soft tissue 2 mm	2.84 ± 0.81	

Facial soft tissue 5 mm clinical	Baseline facial soft tissue 5 mm	2.26 ± 0.65	0.030
	4 months facial soft tissue 5 mm	3.00 ± 1.08	

Ridge width 2 mm with CBCT	Baseline ridge width 2 mm	7.93 ± 0.99	0.002
	4 months ridge width 2 mm	6.46 ± 1.21	

Ridge width 5 mm with CBCT	Baseline ridge width 5 mm	9.06 ± 0.97	0.002^§^
	4 months ridge width 5 mm	7.82 ± 0.87	

Vertical bone height changes in the buccal bone plate by CBCT	Before dental extraction	12.16 ± 1.87	0.368
	Four months after dental extraction	11.88 ± 1.88	

Vertical bone height changes in the palatal bone plate by CBCT	Before dental extraction	12.21 ± 2.32	0.762
	Four months after dental extraction	11.94 ± 2.41	

^∗^Paired sample *t*-test. ^§^Related-Samples Wilcoxon Signed Rank Test.

**Table 4:** Comparing the clinical and tomographical variables between the study and control groups after 4 months from the initial surgery.

Measurements	Group	Mean (mm) ± SD	*p* value^∗^
Mean facial soft tissue changes at 2 mm	Control	−0.77 ± 0.58	0.960
	Study	−0.51 ± 0.36	

Mean facial soft tissue changes at 5 mm	Control	−1.22 ± 1.22	0.303
	Study	−0.73 ± 1.13	

Mean alveolar ridge width changes at 2 mm	Study	−1.47 ± 1.22	0.003^§^
	Control	−3.54 ± 1.26	

Mean alveolar ridge width changes at 5 mm	Study	−1.23 ± 0.87	0.016^∗^
	Control	−2.24 ± 1.06	

Mean changes of the buccal bone plate height	Study	−0.84 ± 0.83	0.005^∗∗^
	Control	−0.31 ± 0.11	

Mean changes of the palatal bone plate height	Study	−0.71 ± 0.82	0.043^§^
	Control	−0.27 ± 0.23	

^∗^Paired sample *t*-test. ^§^Related-Samples Wilcoxon Signed Rank Test.

**Table 5:** Descriptive and comparative statistics of the histomorphometric parameters of the newly formed bone.

Area	Group	Mean (mm^2^) ± SD	*p* value^∗^
Trabeculae bone area	Control	0.72 ± 0.23	0.0001
	Study	1.33 ± 0.24	

Bone marrow area	Control	1.12 ± 0.23	0.0001^§^
	Study	0.49 ± 0.19	

Total bone area	Control	2.20 ± 0.48	0.330
	Study	2.38 ± 0.49	

^∗^Independent sample *t*-test. ^§^Mann–Whitney *U* Test.

## Data Availability

The XLE. Sheets of data used to support the findings of this study are available from the corresponding author upon request.
